# Genome-wide association and systems genetic analyses of residual feed intake, daily feed consumption, backfat and weight gain in pigs

**DOI:** 10.1186/1471-2156-15-27

**Published:** 2014-02-17

**Authors:** Duy Ngoc Do, Tage Ostersen, Anders Bjerring Strathe, Thomas Mark, Just Jensen, Haja N Kadarmideen

**Affiliations:** 1 Section of Animal Genetics, Bioinformatics and Breeding, Department of Veterinary Clinical and Animal Sciences, Faculty of Health and Medical Sciences, University of Copenhagen, 1870 Frederiksberg C, Denmark; 2 Danish Agriculture & Food Council, Pig Research Centre, Axeltorv 3, Copenhagen V, 1609, Denmark; 3 Department of Molecular Biology and Genetics, Aarhus University, Blichers Alle 20, 8830 Tjele, Denmark

**Keywords:** GWAS, Residual feed intake, Backfat, Pigs, Systems genetics, Pathways

## Abstract

**Background:**

Feed efficiency is one of the major components determining costs of animal production. Residual feed intake (RFI) is defined as the difference between the observed and the expected feed intake given a certain production. Residual feed intake 1 (RFI1) was calculated based on regression of individual daily feed intake (DFI) on initial test weight and average daily gain. Residual feed intake 2 (RFI2) was as RFI1 except it was also regressed with respect to backfat (BF). It has been shown to be a sensitive and accurate measure for feed efficiency in livestock but knowledge of the genomic regions and mechanisms affecting RFI in pigs is lacking. The study aimed to identify genetic markers and candidate genes for RFI and its component traits as well as pathways associated with RFI in Danish Duroc boars by genome-wide associations and systems genetic analyses.

**Results:**

Phenotypic and genotypic records (using the Illumina Porcine SNP60 BeadChip) were available on 1,272 boars. Fifteen and 12 loci were significantly associated (*p* < 1.52 × 10^-6^) with RFI1 and RFI2, respectively. Among them, 10 SNPs were significantly associated with both RFI1 and RFI2 implying the existence of common mechanisms controlling the two RFI measures. Significant QTL regions for component traits of RFI (DFI and BF) were detected on pig chromosome (SSC) 1 (for DFI) and 2 for (BF). The SNPs within *MAP3K5* and *PEX7* on SSC 1, *ENSSSCG00000022338* on SSC 9, and *DSCAM* on SSC 13 might be interesting markers for both RFI measures. Functional annotation of genes in 0.5 Mb size flanking significant SNPs indicated regulation of protein and lipid metabolic process, gap junction, inositol phosphate metabolism and insulin signaling pathway are significant biological processes and pathways for RFI, respectively.

**Conclusions:**

The study detected novel genetic variants and QTLs on SSC 1, 8, 9, 13 and 18 for RFI and indicated significant biological processes and metabolic pathways involved in RFI. The study also detected novel QTLs for component traits of RFI. These results improve our knowledge of the genetic architecture and potential biological pathways underlying RFI; which would be useful for further investigations of key candidate genes for RFI and for development of biomarkers.

## Background

Residual feed intake (RFI), defined as the difference between the observed feed intake and the predicted feed intake based on average daily gain and backfat, is a sensitive and accurate indicator for feed efficiency in livestock [1]. Selection for reduced RFI can improve the efficiency of energy utilization without reducing the feed intake capacity that is required for production [2]. Recent studies showed lower RFI selection resulted in better feed conversion efficiency and meat quality in pigs [3] and lower environmental impact in pigs [4], sheep [5] and cattle [6]. Therefore, selection for reduced RFI is important for both economic and environmental aspects of production. Residual feed intake has moderate heritabilities (0.34-0.38) in Danish Duroc pigs and responds to selection [7].

The understanding of genetic mechanisms underlying traits could potentially be important for setting up priors for (genetic) variances in genomic selection or help finding candidate genes for marker- or gene-assisted selection [8,9]. Many approaches including linkage analyses, genome-wide association studies (GWAS), candidate gene association and transcriptomic profiling for RFI have been performed to unravel the genetic background behind the complex trait in many species. For instance, GWAS and linkage studies were performed by [10-12], candidate gene approaches were carried out in [13-19] or transcriptomic studies were used in cattle [20]. These studies revealed many candidate genes and offer background information for genetic studies of RFI in cattle. Compared to cattle, fewer genomic studies have been conducted for RFI in pigs. Gilbert *et al*. [21] detected a QTL on pig chromosome (SSC) 5 and 9 for RFI in growing pigs in a Piétrain–Large White backcross. Fat *et al*. [22] indicated SNPs in the *FTO* and *TCF7L2* gene were significantly associated with RFI in a candidate gene study. Using transcriptomic approaches, Lkhagvadorj *et al*. [23] found many genes in fat (311) and in liver (147) that were differently expressed in low and high RFI pigs in response to caloric restriction and indicated that lipid metabolic pathways was important for regulation of RFI. A recent GWAS has revealed several major QTLs on SSC 7 and 14 influencing RFI in Yorkshire pigs [24]. Jiao *et al*. [25] detected a significant region for FCR on SSC 4 but did not find any significant QTL for RFI in 1,022 Duroc boars. Sahana *et al*. [26] performed GWAS for FCR and found a significant QTL for the trait on SSC 14 in Duroc pigs. Feed conversion ratio is closely related to RFI and is currently included in the selection index for the Danish pig breeds. However, ratio traits such as FCR are not ideal for statistical and biological reasons [27] and there is still a debate about what exactly is the best definition for feed efficiency in production animals. RFI is not ideal under all circumstances either [2], but are well established and increasingly used as a measure for animal breeding and selection in all livestock species. Also, for the purpose of understanding the biology behind feed efficiency it was preferred to have a measure which is independent of daily gain. Therefore, the GWAS and systems genetics study was performed on RFI and its component traits to identify genetic variants and potential candidate genes for these traits as well as possible biological mechanism controlling feed efficiency in Duroc boars.

## Methods

### Estimation of residual feed intake and deregressed estimated breeding values

All phenotypic data used in this study of 7,358 Duroc pigs were recorded at a central test station (Bøgildgård) for a period of 4 years (2008 to 2011) and were supplied by the Pig Research Centre, Danish Agriculture and Food Council [7]. Pigs were fed ad libitum from 30 kg to approximately 100 kg BW with the same feed composition. Feed intake was recorded by ACEMA64 (ACEMO, Pontivy, France) automatic dry feeding stations. All data records in test station including the amount of food in each visits, number of visit to feeder per day and time spent in each visit were sent to Pig Research Center for further processes. The boars were weighted by the technical staff on arrival and at regular intervals (~twice a month) during the growth period. The methods of calculation of RFI have been discussed in [7]. In summary, residual feed intake was computed as the difference between the observed average daily feed intake and the predicted daily feed intake. Two models were used to define RFI. In the first model for RFI1, predicted daily feed intake was estimated using linear regression of DFI on initial test weight (BWd) and average daily gain from 30 kg to 100 kg (ADG). In the second model for RFI2, BWd, ADG and backfat were used as regressors. The two measures of RFI are closely related with an overall phenotypic correlation of 0.96 in Duroc pigs [7]. The estimated breeding values (EBVs) for RFI and these component traits were calculated by single-trait animal model with fixed effect of herd-week-section, and random effects of pen, additive genetics and residuals [7]. These EBVs were calculated using Best Linear Unbiased Predictions [28] based on pedigree traced back to 1970, including 34 generations. Deregressed estimated breeding values (dEBVs) were used as dependent variable in GWAS. The deregression procedure of Garrick *et al.*[29] was used. It adjusts for ancestral information, such that the dEBV only contains their own and the descendant’s information on each animal. Deregressed EBVs have unequal variances and therefore, should be used in a weighted analysis. The weight for the i^th^ animal was estimated as:

$$w_{i}=\left(1-h^{2}\right)/\left[\left(c+\frac{\left(1-r_{i}^{2}\right)}{r_{i}^{2}}\right)h^{2}\right],$$

in which c was the part of the genetic variance that was assumed to be not explained by markers (c = 0.1), h^2^ was the heritability of the trait, and $r_{i}^{2}$ was the reliability of the dEBV of the i^th^ animal. Summary of raw phenotypes, dEBV and weight factors of dEBV for RFI and its component traits of genotyped animals is shown in Table 1.

**Table 1:** **Statistical description for residual feed intake and its component traits of genotyped animals used in the study**^**1**^

**Traits**^**2**^	**Phenotype**	**dEBV**	**Reliability of dEBV**	**Weight factors for dEBV**
DFI(kg)	2.29 ± 0.31	−0.20 ± 0.41	0.18 ± 0.08	0.64 ± 0.54
ADG(kg/day)	1.10 ± 0.10	0.10 ± 0.13	0.19 ± 0.08	0.60 ± 0.47
BF(mm)	7.69 ± 1.04	0.53 ± 0.06	0.43 ± 0.08	1.01 ± 0.58
RFI1(kg)	−0.08 ± 0.30	−0.20 ± 0.25	0.24 ± 0.09	1.07 ± 0.96
RFI(g)	−0.08 ± 0.29	−0.20 ± 0.24	0.25 ± 0.09	1.13 ± 0.99

^1^: The mean residual feed intake was calculated from only genotyped animals. The mean of RFI in the whole population was zero. The negative mean values from current data set implied that these animals have better feed efficiency. The values are shown as mean ± SD.

^2^: DFI: Daily feed intake, ADG: Average daily gain, BF: Backfat, RFI1: Residual feed intake 1, RFI2: Residual feed intake 2, dEBV: Deregressed EBV.

### Genotyping and SNP data validation

The details of the genotyping method have been described previously [30,31]. In summary, genomic DNA was isolated from all specimens by treatment with proteinase K followed by sodium chloride precipitation and SNPs were genotyped on the PorcineSNP60 Illumina iSelect BeadChip. The criteria for screening the genomic data was a call rate per animal of 0.95, call rate per SNP marker of 0.95, Hardy Weinberg equation test with p < 0.0001, and minor allele frequency > 0.05.

### Statistical analyses

#### Linear mixed model used for genome wide association studies

To control the false positives due to family structure, the following linear mixed model was used to analyze the association between the SNP (modeled individually; one at a time) and the phenotypes:

y=1µ+Za+mg+e

where y is the vector of dEBVs for RFI (also for other phenotypes including ADG, DFI and BF), **1** is a vector of 1 s with length equal to number of observations, μ is the general mean, Z is an incidence matrix relating phenotypes to the corresponding random polygenic effect, a is a vector of the random polygenic effect ~ $N\left(0,A\sigma_{u}^{2}\right)$, where A is the additive relationship matrix and $\sigma_{u}^{2}$ is the polygenic variance, m is a vector with genotypic indicators (−1, 0, or 1) associating records to the marker effect, g is a scalar of the associated additive effect of the SNP, and e is a vector of random environmental deviates ~ $N\left(0,{W^{-1}}_{e}^{2}\right)$, where $\sigma_{e}^{2}$ is the general error variance and W is the diagonal matrix containing weights of the dEBVs. The model was analysed by restricted maximum likelihood (REML) using the DMU software [32] and testing was done using a Wald test against a null hypothesis of g = 0. The genome-wide significant association following Bonferroni multiple testing correction at 5% significant level was a p value of 1.52×10^-6^. The Bonferroni correction is highly conservative and may result in too stringent a threshold and hence many false negative results [33]. Therefore, we also considered a more liberal significant threshold where a SNP was considered to have moderate or suggestive significant association with p < 5×10^-5^[34]. Both significant and suggestive SNPs were used in bioinformatics analysis.

### Detection of linkage disequilibrium block and haplotypes

Linkage disequilibrium (LD) block analyses were performed for the chromosomal regions with multiple significant SNPs clustered. The blocks were defined using Haploview [35] with the criteria suggested by Gabriel *et al.*[36] to further characterize candidate regions affecting RFI. The criteria by Gabriel *et al.*[36] defined a haplotype block as a region over which 95% of informative SNP pairs show strong LD (strong LD is defined if the one-sided upper 95% confidence bound on D′ is > 0.98 and the lower bound is above 0.7)

### Systems genetics analyses

SNP positions were updated according to the newest release from Ensembl (Sscrofa10.2 genome version). Identification of the closest genes to significant and suggestive SNPs was obtained using Ensembl annotation of Sscrofa10.2 genome version (http://ensembl.org/Sus_scrofa/Info/Index). The positional candidate genes within 1 Mb bin size of significant and suggestive SNPs were scanned using function *GetNeighGenes()* in the NCBI2R R-package at http://cran.r-project.org/web/packages/NCBI2R/index.html[37]. Investigation of functional categories and the relevant KEGG pathways for the genes within 1 Mb bin size of significant SNPs was performed using the Database for Annotation, Visualization and Integrated Discovery (DAVID) available at http://david.abcc.ncifcrf.gov/[38]. The selection of 1 Mb bin size or 0.5 Mb flanking regions of significant SNPs was based on previous results of Sahana *et al*. [26] who observed very high LD in this Duroc pig population (average r^2^ = 0.3 between two markers in 1 Mb distance). This result suggests a similar distance (1 Mb bin size) can be used to capture the causal genes/SNPs. The positional candidate genes identified by NCBI2R package were first assigned to the KEGG pathways (http://www.genome.jp/kegg/pathway.html) and GO terms (http://www.geneontology.org/). Then, these related pathways/GO terms were tested if they appear by random chance by using modified Fisher exact test. The pathway/GO terms were declared to be important for the traits if they do not appear by a random chance with p < 0.05 [38].

## Results

### Genome wide association results for residual feed intake

**Figure 1:**
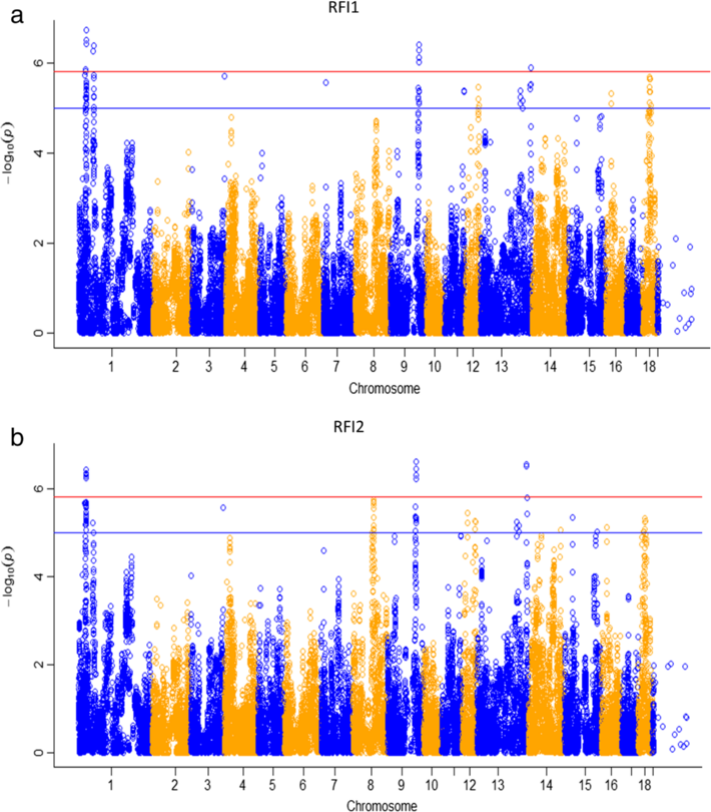
**Manhattan plot of genome-wide *p-*values of association for residual feed intake.** Legends: **(a)**: Manhattan plot for Residual feed intake 1 (RFI1), **(b)**: Manhatan plot for Residual feed intake 2 (RFI2). The horizontal red and blue lines represent the genome-wide significance threshold at p < 1.52 × 10^-6^ and *p* < 5 × 10^-5^, respectively.

Following quality control, 23,795 markers were first excluded as having a low (<5%) minor allele frequency, secondly 1,836 markers were excluded because of low (<95%) call rate and finally 3,463 markers were excluded because they were not in HWE (p < 0.0001), two animals were removed because of low (<95%) call rate. A final set of 33,241 SNPs and 1,272 pigs was retained for GWAS. Fifteen and twelve SNPs were significantly associated to RFI1 and RFI2 at p < 1.52 × 10^-6^ (Bonferroni correction), respectively in which nine SNPs associated with both traits (Tables 2 and 3). The highest contribution of a significant SNP to additive genetic variance was only 0.21% in each trait. Moreover, 138 and 176 SNPs were found to have suggestive (moderately significant) association with RFI1 and RFI2 at p < 5 × 10^-5^, of which 124 SNPs have been found to be associated with both traits. High numbers of significant SNPs for both RFI were found on SSC 1, 8, 9, 11, 12 ,13 ,14, 15, 16 and 18, while none of them were found on SSC 2, 10 and 17. Several regions: 27-33 Mb on SSC 1, 89-91 Mb on SSC 8, 119-122 Mb on SSC 9 and 26-35 Mb on SSC 18 harbored many associated SNPs for both RFI. Genome wide Manhattan plots displaying the GWA results with the respect to their position are shown in Figure 1a and b. Lists of genes located within 0.5 Mb window from the significant and suggestive SNPs is provided in Additional file 1.

**Table 2:** The significant SNPs associated with residual feed intake 1 (RFI1)

**SNP**	**SSC**^**1**^	**Position**	**p-value**	**MAF**^**2**^	**Var.Exp**^**3**^	**Nearest gene**	**Gene name**
MARC0013869	0	0	5.13 × 10^-7^	0.47	0.21		
MARC0112693	1	30734120	1.52 × 10^-6^	0.44	0.19	*PEX7*	PEX7 peroxisomal biogenesis factor 7
H3GA0001223	1	30,769,583	1.41 × 10^-6^	0.44	0.19	*PEX7*	PEX7 peroxisomal biogenesis factor 7
ASGA0082396	1	30,941,797	3.73 × 10^-7^	0.45	0.20	*MAP3K5*	mitogen-activated protein kinase kinase kinase 5
ALGA0106992	1	30,962,276	3.13 × 10^-7^	0.45	0.20	*MAP3K5*	mitogen-activated protein kinase kinase kinase 5
ASGA0094502	1	30,978,281	3.73 × 10^-7^	0.45	0.20	*MAP3K5*	mitogen-activated protein kinase kinase kinase 5
ALGA0107451	1	31,008,523	3.07 × 10^-7^	0.45	0.20	*MAP3K5*	mitogen-activated protein kinase kinase kinase 5
H3GA0001228	1	31,202,546	1.89 × 10^-7^	0.44	0.21	*MAP3K5*	mitogen-activated protein kinase kinase kinase 5
ALGA0003540	1	60,869,380	5.44 × 10^-7^	0.15	0.15	*NT5E*	5'-nucleotidase, ecto
ALGA0003690	1	64,094,344	4.04 × 10^-7^	0.26	0.18	*GABRR2*	gamma-aminobutyric acid (GABA) A receptor, rho 2
ALGA0108119	9	120,773,379	5.10 × 10^-7^	0.47	0.21	*ENSSSCG00000022338*	Uncharacterized protein
ALGA0054579	9	120,972,491	3.86 × 10^-7^	0.47	0.21	*ENSSSCG00000022338*	Uncharacterized protein
DRGA0009690	9	121,359,360	9.48 × 10^-7^	0.47	0.20	*ENSSSCG00000022338*	Uncharacterized protein
H3GA0028049	9	121,407,081	7.61 × 10^-7^	0.48	0.20	*ENSSSCG00000022338*	Uncharacterized protein
H3GA0038097	13	21,3691,291	1.29 × 10^-6^	0.12	0.13	*DSCAM*	Down syndrome cell adhesion molecule

^1^: Pig Chromosome ; ^2^: Minor Allele Frequency ; ^3^: Additive variance explained by SNP.

**Table 3:** Significant SNPs associated with residual feed intake 2 (RFI2)

**SNP**	**SSC**^**1**^	**Position**	**p-value**	**MAF**^**2**^	**Var.exp**^**3**^	**Nearest gene**	**Gene name**
MARC0013869	0	0	3.15 × 10^-7^	0.474	0.20		
ASGA0082396	1	30,941,797	5.64 × 10^-7^	0.448	0.19	*MAP3K5*	Mitogen-activated protein kinase kinase kinase 5
ALGA0106992	1	30,962,276	4.68 × 10^-7^	0.448	0.19	*MAP3K5*	Mitogen-activated protein kinase kinase kinase 5
ASGA0094502	1	30,978,281	5.09 × 10^-7^	0.449	0.19	*MAP3K5*	Mitogen-activated protein kinase kinase kinase 5
ALGA0107451	1	31,008,523	4.54 × 10^-7^	0.450	0.19	*MAP3K5*	Mitogen-activated protein kinase kinase kinase 5
H3GA0001228	1	31,202,546	3.67 × 10^-7^	0.443	0.20	*MAP3K5*	Mitogen-activated protein kinase kinase kinase 5
ALGA0108119	9	120,773,379	3.64 × 10^-7^	0.473	0.20	*ENSSSCG00000022338*	Uncharacterized protein
ALGA0054579	9	120,972,491	2.49 × 10^-7^	0.473	0.20	*ENSSSCG00000022338*	Uncharacterized protein
DRGA0009690	9	121,359,360	6.02 × 10^-7^	0.475	0.20	*ENSSSCG00000022338*	Uncharacterized protein
H3GA0028049	9	121,407,081	5.02 × 10^-7^	0.475	0.20	*ENSSSCG00000022338*	Uncharacterized protein
ASGA0089950	13	210,531,047	2.87 × 10^-7^	0.113	0.13	*HLSC*	Holocarboxylase synthetase
ASGA0097399	13	210,534,054	3.07 × 10^-7^	0.114	0.13	*HLSC*	Holocarboxylase synthetase

^1^: Pig Chromosome ; ^2^: Minor Allele Frequency ; ^3^: Additive variance explained by SNP.

### Genome wide association results for component traits of residual feed intake

**Figure 2:**
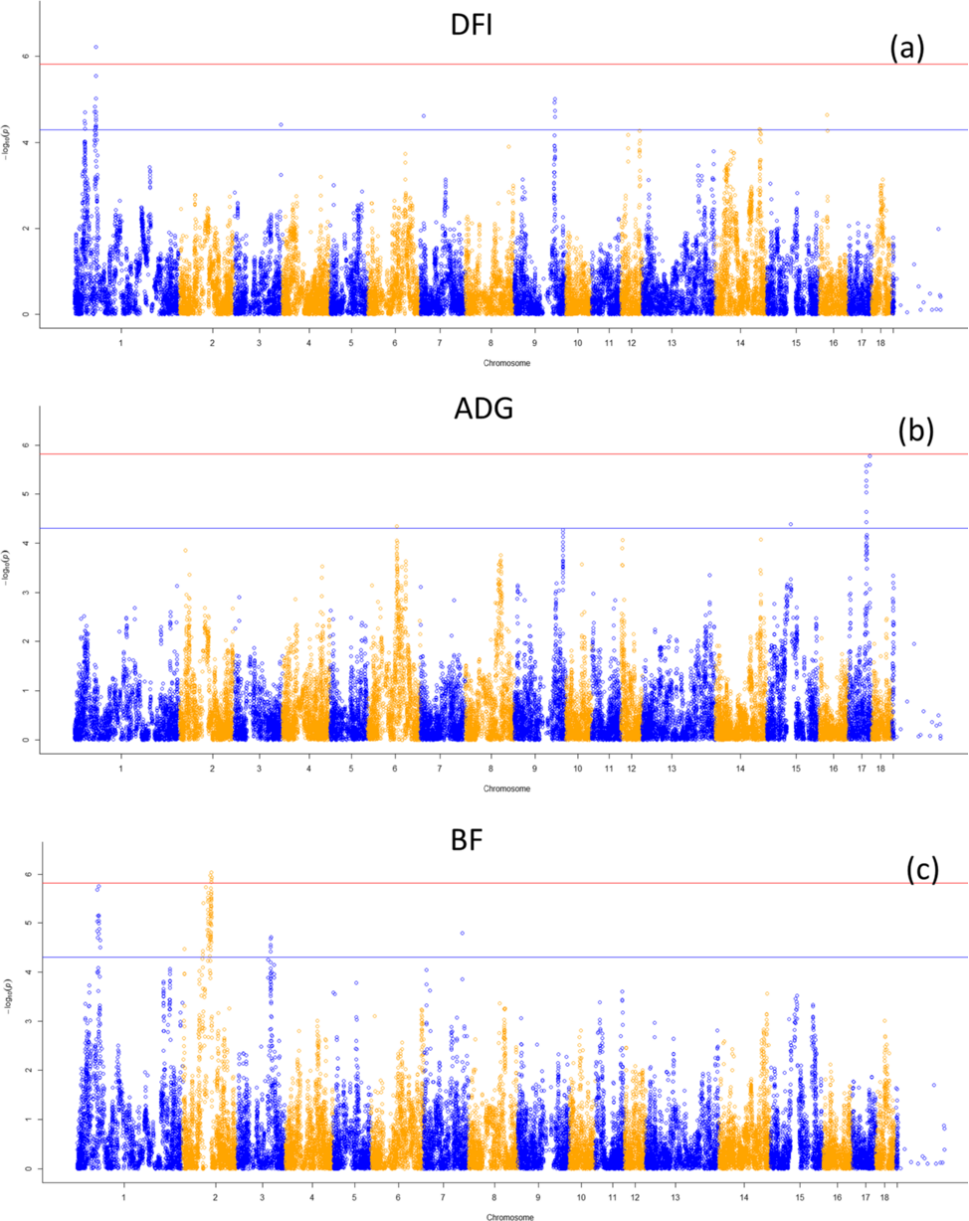
**Manhattan plot of genome-wide *p*-values of association for component traits of residual feed intake.** Legends: **(a)**: Manhattan plot for Daily feed intake (DFI), **(b)**: Manhatan plot for Average daily gain (ADG), **(c)**: Manhatan plot for backfat (BF). The horizontal red and blue lines represent the genome-wide significance threshold at *p* < 1.52 × 10^-6^ and *p* < 5 × 10^-5^, respectively.

The genome wide association analysis showed only one significantly associated SNP (p = 6.10 × 10^-7^) for DFI at 64 Mb position on SSC 1 (Table 4). Moreover, 25 other suggestive associated SNP were also found on SSC 1, 3, 7, 9, 14, and 16 and two suggestive SNPs have not yet been mapped on any chromosome (Additional file 1). None of significant SNP was found for ADG, however, 15 suggestive SNPs were identified on SSC 6, 15 and 17 (Additional file 1). Thirteen of them were located in 53–54 Mb on SSC17 and marker ASGA0077977 was tightest association with trait at p = 1.67 × 10^-6^ (Table 4). All of six significant SNPs associated with BF were located on a region of 82-86 Mb on SSC 2 (Table 4). Moreover, 73 suggestive SNPs for BF were also located in the same region (Additional file 1). Fifteen suggestive SNP for BF were located on region of 60-68 Mb on SSC 1 and 7 SNPs was not mapped onto any chromosome. Genome wide Manhattan plots displaying the GWA results for DFI, ADG and BF with the respect to their positions are shown in Figure 2a, b and c, respectively.

**Table 4:** The significant SNPs associated with component traits of residual feed intake

**Traits**^**1**^	**SNP**^**2**^	**SSC**^**3**^	**Pos**	**MAF**	**p-value**	**Nearest gene**	**Gene name**
DFI	ALGA0003690	1	64,094,344	0.28	6.10 × 10^-7^	*GABRR2*	Gamma-aminobutyric acid (GABA) A receptor, rho 2
ADG*	ASGA0077977	17	63,740,625	0.32	1.67 × 10^-6^	*CBLN4*	Cerebellin 4 precursor
BF	ALGA0014061	2	84,789,103	0.07	9.06 × 10^-7^	*ENSSSCG00000024586*	Novel gene
BF	ALGA0014028	2	82,276,435	0.08	1.06 × 10^-6^	*RGS14*	Regulator of G-protein signaling 14
BF	ASGA0010625	2	86,139,077	0.08	1.17 × 10^-6^	*COL4A3BP*	Collagen type IV alpha-3-binding protein
BF	ALGA0014098	2	85,830,426	0.07	1.27 × 10^-6^	*ANKRD31*	Ankyrin repeat domain 31
BF	DRGA0003063	2	85,710,507	0.07	1.45 × 10^-6^	*GCNT4*	Glucosaminyl (N-acetyl) transferase 4, core 2
BF	MARC0074327	2	85,539,255	0.07	1.46 × 10^-6^	*FAM169A*	Family with sequence similarity 169, member A

^1^: DFI: Daily feed intake, ADG: Average daily gain, BF: Backfat ^2^: Pig Chromosome ; ^3^: Minor Allele Frequency.

*: The tighest associated SNP for ADG have not pass GW significant thresold (1.52 × 10^-6^).

### LD block, haplotype analysis and functional categories of positional candidate genes for residual feed intake

**Figure 3:**
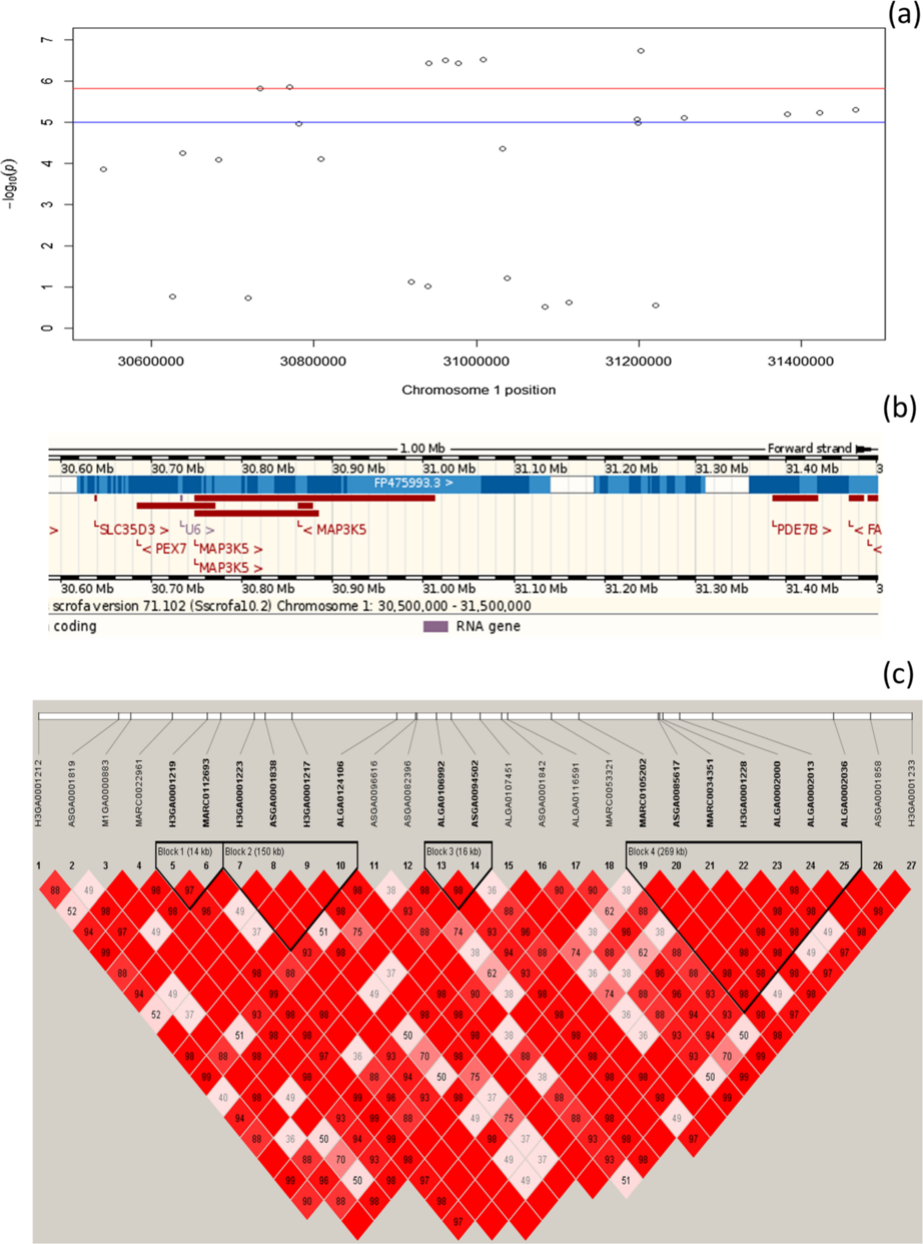
**Linkage disequilibrium block and Ensembl genes on region from 30.5 to 31.5 Mb on pig chromosome 1. Legends: (a)**: Manhattan plot of genome-wide p values for region from 30.5 to 31.5 Mb on pig chromosome 1, **(b)**: Ensembl genome regions from 30.5 to 31.5 Mb on SSC 1, **(c)**: Linkage disequilibrium block detected in the region, markers in blocks shown in bold.

**Figure 4:**
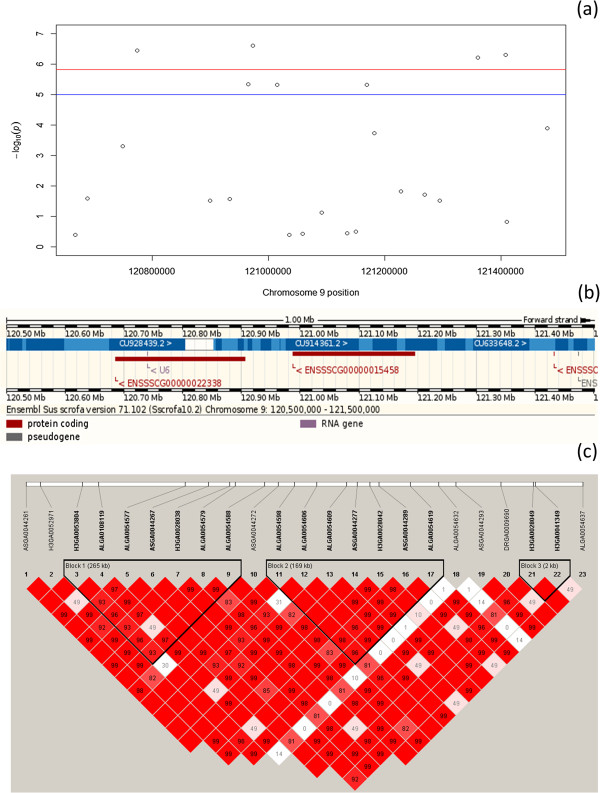
**Linkage disequilibrium block and Ensembl genes in the region from 120.5 to 121.5 Mb on pig chromosome 9.** Legends: **(a)**: Manhattan plot of genome-wide *p-*values for RFI 1 in the region from 120.5 to 121.5 Mb on pig chromosome 9, **(b)**: Ensembl genome region from 30.5 to 31.5 Mb on pig chromosome 9, **(c)**: Linkage disequilibrium block detected in the region, markers in these blocks were shown in bold.

Four and three LD blocks were identified in regions 30.5-31.5 Mb on SSC 1 and 120.5-121.5 Mb on SSC 9, respectively. The Manhattan plot, LD blocks and Ensembl genome for candidate regions on SSC 1 and SSC 9 were shown in Figures 3 and 4, respectively. Frequency of each haplotype for different LD blocks on SSC 1 and SSC 9 was shown in Additional files 2 and 3, respectively. On chromosome 1, each LD block has similar frequency for major haplotypes with frequency ranging from 0.44 to 0.55. On chromosome 9, haplotype 2112212 for LD block 1 and haplotype 2121112 for LD block 2 appeared more often than other haplotypes (1 is minor allele and 2 is major allele).

Due to high number of common SNPs for both RFI1 and RFI2, we decided to use positional candidate genes found for significant/suggestive SNPs for RFI2 for functional annotation. There were, 619 positional candidate genes for RFI2 to these significant/suggestive SNPs and were used as input in DAVID (Additional file 1). The functional annotation of positional candidate genes based on biological process and KEGG pathways involving in RFI2 is shown in Tables 5 and 6, respectively. The GO termed regulation of protein metabolic process, cellular lipid metabolic process and lipid metabolic process showed significant overrepresentation of genes statistically associated with RFI (p < 0.05). The gap junction, phosphatidylinositol signaling system inositol phosphate metabolism and insulin signaling pathways were statistically associated with RFI (p < 0.05).

**Table 5:** **The results of Gene Ontology (GO) analysis including genes in 0.5 Mb flanking size to SNPs with p < 5.0 × 10**^**-5**^

**Go sub-ontology**	**GO term accession**	**GO term description**	**Involved genes**	**David p-value**
Biological process	GO:0051246	Regulation of protein metabolic process	*UBE2L3, PRKAR1A, UBE2J1*	0.03
Biological process	GO:0044255	Cellular lipid metabolic process	*SGMS1, PTEN, ALOX12, PRKAG3*	0.04
Biological process	GO:0006629	Lipid metabolic process	*SGMS1, PTEN, ALOX12, PRKAG3, PLCD4*	0.05

**Table 6:** **Results of functional annotation on KEGG pathway including genes 0.5 Mb flanking size to SNPs with p < 5.0 × 10**^**-5**^

**Term**	**Pathway name**	**Number of involved genes**	**Involved genes**	**David p-value**
ssc05213	Endometrial cancer	5	*TCF7L2,ERK2, PTEN, PIK3R5. TP53*	0.001
ssc04540	Gap junction	6	*TUBA4A, ERK2, ADRB1, PRKG1, TUBA3D, TUBA1A*	0.002
ssc04070	Phosphatidylinositol signaling system	4	*PTEN, PIK3R5, PLCD4, PI4KA*	0.013
ssc00562	Inositol phosphate metabolism	4	*PTEN, PIK3R5, PLCD4, PI4KA*	0.015
ssc05214	Glioma	4	*ERK2, PTEN, PIK3R5, TP53*	0.026
ssc05215	Prostate cancer	5	*ERK2, PTEN, PIK3R5, TP53, TCF7L2*	0.027
ssc04910	Insulin signaling pathway	5	*ERK2, SLC2A4, PIK3R5, PRKAR1A, PRKAG3*	0.031
ssc05218	Melanoma	4	*ERK2, PTEN, PIK3R5. TP53*	0.033

## Discussion

### QTLs, LD blocks and candidate genes for residual feed intake

Despite differences in the estimation models, RFI had very high genetic correlation with each other (r_g_ = 0.96 [7]). Hence it is not surprising that the GWAS results for RFI1 and RFI2 show highly similar genetic architecture (numbers of top SNPs and their genomic positions). Many significant SNPs for both RFI were located in the same genomic regions on SSC 1, 9 and 13, and approximately 80% of suggestive SNPs (124 SNPs) were also found to be associated with both traits. Likewise, Nkrumah *et al*. [39] also reported many concordant QTLs between RFI based phenotype (RFIp) and RFI based genotype (RFIg) in cattle. These authors detected 14 common and 3 distinct QTLs for the two RFI measures. Their high genetic similarity makes it difficult to find unique QTL and candidate genes for each trait.

Two most interesting chromosomal regions for RFI were 30.5-31.5 Mb on SSC 1 and 120.5-121.5 Mb on SSC 9. Seven and five highly significant SNPs for RFI1 and RFI2 were found in chromosomal regions 30.5-31.5 Mb on SSC 1. *MAP3K5* (mitogen-activated protein kinase 5) gene, located from 30,747 to 31,011 kb on SSC 1, might be an interesting candidate gene. *MAP3K5*, also known as apoptosis signal-regulating kinase 1 (*ASK1*), acts as an essential component of the mitogen-activated protein kinase signal transduction pathway in humans [40], it mediates signaling for determination of cell fate such as differentiation and survival in mice [41]. The effect of *MAP3K5* (or in generally, MAPK) on controlling feed intake or RFI may be mediated by variety of pathways such as hormones and growth factors that act through receptor tyrosine kinases (e.g. insulin, epidermal growth factor (EGF) [42]), cytokine receptors (e.g. growth hormone) to vasoactive peptides acting through G protein-coupled, seven-transmembrane receptors (e.g. endothelin) and so on [43]. In cattle, the majority of up-regulated genes in low RFI beef was stimulated by MAPKs [20]. Functional approaches to validate *MAP3K5* as a candidate gene for RFI in pigs is necessary.

The LD block helps to get more detail in QTL regions because several significant/suggestive SNPs were found in the same LD block. Therefore, it could imply that the causative mutation might be in these blocks. These approaches have been extensively applied in many species [31,44,45]. It is also worthy to note that two candidate SNPs (ALGA0106992 and ALGA0094502) are tightly linked (D’ = 0.98) in the LD block 3 (Figure 3). Functional validation for *MAP3K5* as a candidate gene for RFI needs to also take into account the different haplotypes and linkage phases. Close to *MAP3K5* gene, the variant MARC0112693 was also significantly associated with RFI. The variant was located in the intronic region on *PEX7* gene, which encodes the cytosolic receptor for the set of peroxisomal matrix enzymes targeted to the organelle by the peroxisome targeting signal 2. The gene plays an essential role in peroxisomal protein import and defects in this gene cause peroxisome biogenesis disorders (PBDs), which are characterized by multiple defects in peroxisome function in human [46]. Moreover, the chromosomal region is homologous with human chromosomal region (HSA) 6q.23. The HSA6q.23 contained *MAP3K5*–PEX7 haplotype block which was found associated with age at onset in Huntington's disease [47,48] and with modulation of fetal hemoglobin levels in sickle cell anemia [49]. Therefore, RFI might not be controlled by a single gene but by LD block in the region.

On chromosome 1, two SNPs were significantly associated with only RFI1. Interestingly, the ALGA0003690 polymorphism were also found to be significantly associated with DFI in the same population [31]. The mutation is located in intronic region of *GABRR2* gene. The gene encodes a receptor of gamma-aminobutyric acid (GABA), the major inhibitory neurotransmitter in the vertebrate brain. Therefore, *GABRR2* might be an interesting candidate gene for both daily feed intake and RFI1. Another possible candidate gene for RFI1 is *NT5E*, which encodes for a protein that catalyzes the conversion of extracellular nucleotides to membrane-permeable nucleosides in human [50]. Due to *NT5E* using AMP as a substrate, the involvement of their gene with residual feed intake might be via energy balance.

The second interesting region for RFI is 120.5-121.5 Mb on SSC 9. Four highly significant SNPs for RFI1 and three for RFI2 were found in the region. The SNPs were located in all different LD blocks (Figure 3), and were highly linked to several suggestive SNPs (Additional files 2 and 3). However, both of these nearest genes found in the region have not been functionally studied.

A significant SNP for RFI1 and two significant SNPs for RFI2 were found on SSC 13. Because these SNPs are very closely located to each other, the region might contain a QTL for both traits. Notably, a marker H3GA0038097, which was significantly associated with RFI1, was also significantly associated with RFI2 (p = 1.62 × 10^-6^). The nearest gene, *DSCAM* encodes for Down syndrome cell adhesion molecule, which plays a role in neuronal self-avoidance as discovered in a mice model [51]. Recently, Garrett *et al*. [52] reviewed the role of DSCAMs proteins and suggested that their importance of balancing these developmental mechanisms. Close to the *DSCAM* gene, the *HLCS* (holocarboxylase synthetase) gene plays an important role in gluconeogenesis, fatty acid synthesis and branched chain amino acid catabolism in human [53]. Both of *DSCAM* and *HLCS* have not been functionally characterized in pigs, however, since their function involves developmental balance and glucose/fatty acid regulations, they might be important candidate genes for RFI in pigs.

A suggestive QTL spanned a region from 83-92 Mb on SSC 8 and contained five SNPs for RFI1 and 26 suggestively significant SNPs for RFI2 which may be interesting. Sahana et al. [26] also found a significant SNP for FCR in the same region in the same Duroc population. The other suggestive QTL regions are 54-56 Mb on SSC 12 containing eight SNPs for both RFI and 26-36 Mb on SSC 18 containing 24 SNPs for RFI1 and 20 SNPs for RFI2. These QTL regions also contained a number of potential candidate genes for RFI. The QTL on SSC12 for RFI was close to QTL for FCR in Meshan × Large White cross populations previously recorded by [54] and QTL on SSC 18 was coincided with QTL for FCR on chromosome in the genetically diverse founder groups Meishan , Pietrain and European Wild Boar previously by [55]. However, more analyses are needed to confirm if they are true QTL for RFI.

### Systems genetics of daily feed intake, average daily gain and backfat

#### QTLs and candidate genes for daily feed intake

We have identified ALGA0003690 (G/A) significantly associated with DFI based on genotype records from 2008 to 2011 in the same population [31]. Although, we have added 300 genotyped animals (recorded in 2012), we still detected only this SNP passed genome-wide significant threshold in the current study. However, we also detected more suggestive loci on SSC 3, 14 and 16 in the current study. The most interesting putative candidate gene for DFI might be Gamma-aminobutyric acid receptor subunit rho-2 (*GABRR2*) gene. The gene encode for the most important inhibitory neurotransmitter in the vertebrate central nervous system and it plays function in feed/food intake as discussed in [31]. Some other nearest genes may be interesting are G protein pathway suppressor 2 (*GPS2*) gene on SSC 3, alpha-2A receptor gene (*ADRA2A*) on SSC 14 and Nipped-B homolog gene (*NIPBL*) in SSC 16 (Table S). For instance, *ADRA2A* is one of candidate genes for obesity and diabetes [56] and variants in the gene was associated with satiation [57]. Because the pig is a model for human obesity research [31], *ADRA2A* is worthy to functionally investigate.

#### QTLs and candidate genes for average daily gain

Understanding the genomics controlling components traits of RFI helps to better prioritize candidate SNP/genes for further investigation. Amongst suggestive regions found associated with ADG, none of them overlapped with QTL regions detected for RFI. Therefore, markers assisted selection based on candidate genes for RFI (identified in this population) would not have influence on daily gains of pigs. The ASGA007797 marker was tightly associated with ADG (p = 1.67 × 10^-6^) (Table 5) and was located within brain functioned *CBLN4* gene. The gene encodes for new transneuronal cytokines [58] which have been highly involved in insulin secretion in rats [59]. On chromosome 17, we also identified a region from 53.4-54.2 Mb which contains 8 suggestive SNPs for ADG. Several genes in this region might be interesting such as *NCOA5* (Nuclear receptor coactivator 5), *SLC35C2* (Solute carrier family 35, member C2) and *CD40* (TNF receptor superfamily member 5) might interesting for further investigation. Moreover, on chromosome 6, a suggestive SNP was found in QTL regions detected for ADG in several resource populations [60,61]. This SNP was located close to Ras-related GTP binding C (*RRAGC*). *RRAGC* encodes for a members of Rag small GTPases, which regulate the growth-controlling TOR signaling pathway (reviewed in [62]). However, many nearest genes for ADG have not been functionally characterized in pigs.

#### QTLs and candidate genes for backfat

In pigs, backfat is one of the phenotypes that have been studied in many resource populations. The QTLs for BF have been mapped in every pig chromosome. Several GWAS studies have been also performed for BF such as those reported by Fontanesi *et al*. [63]; they reported candidate genes on SSC 7 and 18 associated with Italian heavy pigs. Other studies include Onteru *et al*. [24] who reported fat metabolism genes on SSC 3, 7 and 18 for BF in Yorkshire pigs and Okumura *et al*. [64] who reported QTL on SSC 6 for backfat thickness in Japanese Duroc pig population. Because all significant SNPs (Table 5 and more than 70 suggestive QTLs (Additional file 1) for BF were located in the region of 81-86 Mb on SSC 2, we assumed that a QTL in this region was affecting BF. In this region, many genes have been shown to have important role in fat deposition and lipid metabolism. For instance, 3-hydroxy-3-methylglutaryl-CoA reductase (*HMGCR*) gene spanned from 85,967 to 85,990 kb on SSC 2 which encodes for the well-known enzyme regulating the synthesis of cholesterol in humans and other species. Pigs with divergent backfat thickness also expressed different HMGCR activity in liver [65]. Canovas *et al*. [66] found that the mutation in pig *HMGCR* gene (807A > G) was associated with not only cholesterol but also intramuscular fat content in commercial Duroc pig line. It is also worthy to note that we also detected suggestive effect on BF in the chromosomal region at 63-64 Mb on SSC 1 where a QTL for DFI and RFI was reported. Therefore, the characterization of these regions needs to consider the effects of pleiotropic QTL. Moreover, the suggestive QTL at 116.3 Mb on SSC 7 was also very close to the region where Orteru *et al*. [24] detected QTL for BF at 112 Mb position. *GALA* gene encodes to galactosylceramidase might be interesting candidate gene because its protein (an enzyme) breaks down galactolipids and plays role in lipid metabolism in kidney and epithelial cells of small intestine and colon [67].

#### Inferring pathways and biological functions of nearby genes for residual feed intake

Based on biological function, several nearby genes of significant SNPs for RFI (*SGMS1, PTEN, ALOX12, PRKAG3, PLCD4*) were also clustered in lipid metabolic process (GO:0044255 and GO:0006629) in the current study (Table 5). The relation between lipid metabolism and residual feed intake has been shown in pigs [23] and cattle [68]. Moreover, we also recorded nearby genes involved in regulation of protein metabolic process (*UBE2L3, PRKAR1A, UBE2J1*). *PRKAR1A* encodes for protein kinase A (PKA, aka cAMP-dependent protein kinase) which is involved in the regulation of lipid and glucose metabolism and is a component of the signal transduction mechanism of certain G protein-coupled receptors in humans [69]. Malek *et al*. [70] characterized the porcine prepro-orexin gene and found high linkage among *PRKAR1A, GH1* and *BRCA1* genes. The same authors speculated that *PRKAR1A* is a candidate gene for feed intake. Nevertheless, lipid metabolic process is a very broad term, and therefore it might be worthy to further investigate which sub-terms in the process are involved in RFI metabolism.

Interestingly, we also found that the genes clustered in insulin signaling pathway. In the insulin signaling pathway, insulin binds to its receptor resulting in the tyrosine phosphorylation of insulin receptor substrates (IRS) by the insulin receptor tyrosine kinase (INSR). This allows association of IRSs with the regulatory subunit of phosphoinositide 3-kinase (PI3K). PI3K activates 3-phosphoinositide-dependent protein kinase 1 (*PDK1*), which activates Akt, a serine kinase. Akt in turn deactivates glycogen synthase kinase 3 (GSK-3), leading to activation of glycogen synthase and thus glycogen synthesis (KEGG pathway, term: ssc04910). Several studies also mentioned that insulin signaling pathway plays important roles in controlling residual feed intake in cattle [12,71] and in pigs [72].

Another potentially relevant pathway is gap junction which consists of 6 nearby genes of significant SNPs for RFI (Table 5). Gap junctions contain intercellular channels that allow direct communication between the cytosolic compartments of adjacent cells [73]. Regulation of feed intake is a complex process, which not only happens inside the cells but also in interactions among cells. *PRKG1* is one of the genes involved in gap junction and is also a candidate gene for RFI in cattle [11] and for intramuscular fat content in pigs [74]. Nevertheless, implying pathways based on GWAS data analyses alone needs caution because many other factors can have an effect on the results such as hidden confounders, threshold for significant p-value of SNP from GWAS data, length of flanking region to get gene list, the statistical test methods and so on [75] and a systems biology approach using additional more or less independent data to verify or add information to the findings would be one of the best approaches to profile pathways underlying complex traits [8].

In summary, this study used comprehensive GWAS and pathway approaches to reveal genetic variants, and genes that control feed efficiency (RFI) and the related traits in pigs and possible biological pathways in which these genes are exerting their effects. This study confirmed highly similar genetic mechanisms underlying different measurement of RFI; however, it could not find distinct genetic markers for RFI2. Therefore, including back fat in the RFI models was not important for this particular data and analyses. In the context of genomic selection for feed efficiency, the estimated magnitude and direction of SNP effects could potentially be useful for specifying more informative prior information in genomic prediction/selection models to increase genetic gain.

## Conclusion

This study revealed possible genetic architecture and potential biological pathways for a feed efficiency measure, RFI in pigs. We identified two important genomic regions including 30.5-31.5 Mb on pig SSC 1 and 120.5-121.5 Mb on SSC 9 for RFI. We also conclude that there is a high similarity of genetic architecture between RFI1 and RFI2. Key positional candidate genes have been found: *MAP3K5, PEX7, ENSSSCG00000022338* and *DSCAM* for both RFI measures. We also detected several novel QTLs for other production traits including DFI, ADG and BF that were components of RFI measure. Systems genetic analyses and functional annotation of nearby genes confirmed an important role of insulin signaling pathway in regulation of RFI and revealed some other possible pathways such as gap junction or inositol phosphate metabolism. Therefore, this study offered important knowledge of the potential candidate genes, biomarkers, genetic architecture and biological pathways for feed efficiency measures.

## Abbreviations

EBV: Estimated breeding values; dEBV: Deregressed estimated breeding values; GWAS: Genome-wide association study; LD: Linkage disequilibrium; SNP: Single nucleotide polymorphism; RFI: Residual feed intake; Bp: Base pairs; kb: Kilo base pairs; Mb: Mega base pairs; MAF: Minor allele frequency; QTL: Quantitative trait locus.

## Competing interest

No conflict of interest.

## Authors' contributions

DND did the analysis with the help of HNK, TO and ABS. DND wrote the first draft of the manuscript. HNK conceived and designed this project and provided supervision for DND in all aspects including GWAS, systems genetics and bioinformatics work. JJ and TM provided useful suggestions on analyses. All authors contributed to writing of this manuscript, read and approved the final manuscript.

## Supplementary Material

Additional file 1 Suggestive SNPs associated with RFI and its component traits, respectively. Click here for file

Additional file 2 Frequency of each haplotype for different LD blocks on pig chromosome 1. Click here for file

Additional file 3 Frequency of each haplotype for different LD blocks on pig chromosome 9. Click here for file
