# Supplementary material for: Genome-wide association and systems genetic analyses of residual feed intake, daily feed consumption, backfat and weight gain in pigs
Source: BMC Genet. 2014 Feb 17;15:27. doi: 10.1186/1471-2156-15-27 (PMC3929553; doi:10.1186/1471-2156-15-27)
Supplement: Additional file 2 — Frequency of each haplotype for different LD blocks on pig chromosome 1. [file 1471-2156-15-27-S2.docx]

| **Locus** | **Haplotype^1^** | **Frequency** | **Marker names** |
| --- | --- | --- | --- |
| Block1 | 21 | 0.44 | H3GA0001219, MARC0112693 |
| Block1 | 12 | 0.10 | H3GA0001219, MARC0112693 |
| Block1 | 22 | 0.46 | H3GA0001219, MARC0112693 |
| Block2 | 1211 | 0.02 | H3GA0001223, ASGA0001838 , H3GA0001217, ALGA0124106 |
| Block2 | 1121 | 0.10 | H3GA0001223, ASGA0001838 , H3GA0001217, ALGA0124106 |
| Block2 | 1112 | 0.44 | H3GA0001223, ASGA0001838 , H3GA0001217, ALGA0124106 |
| Block2 | 2222 | 0.44 | H3GA0001223, ASGA0001838 , H3GA0001217, ALGA0124106 |
| Block3 | 12 | 0.45 | ALGA0106992, ASGA0094502 |
| Block3 | 21 | 0.55 | ALGA0106992, ASGA0094502 |
| Block4 | 2111222 | 0.44 | MARC0105202, ASGA0085617, ALGA0107451, H3GA0001228, ALGA0002000, ALGA0002013, ALGA0002036 |
| Block4 | 2222211 | 0.45 | MARC0105202, ASGA0085617, ALGA0107451, H3GA0001228, ALGA0002000, ALGA0002013, ALGA0002036 |
| Block4 | 1122122 | 0.10 | MARC0105202, ASGA0085617, ALGA0107451, H3GA0001228, ALGA0002000, ALGA0002013, ALGA0002036 |

Additional file 3. Detected happlotypes on the region from 30.5-31.5 Mb on pig chromosome 1

^1 :^ 1 is minor allele and 2 is major allele
