# Supplementary material for: Genome-wide association and systems genetic analyses of residual feed intake, daily feed consumption, backfat and weight gain in pigs
Source: BMC Genet. 2014 Feb 17;15:27. doi: 10.1186/1471-2156-15-27 (PMC3929553; doi:10.1186/1471-2156-15-27)
Supplement: Additional file 3 — Frequency of each haplotype for different LD blocks on pig chromosome 9. [file 1471-2156-15-27-S3.doc]

| **Block** | **Haplotype^1^** | **Frequency** | **Marker names** |
| --- | --- | --- | --- |
| Block1 | 2112212 | 0.526 | H3GA0053804, ALGA0108119, ASGA0044277, ASGA0044267, H3GA0028038, ALGA0054579, ALGA0054588 |
| Block1 | 221112 | 0.186 | H3GA0053804, ALGA0108119, ASGA0044277, ASGA0044267, H3GA0028038, ALGA0054579, ALGA0054588 |
| Block1 | 2221222 | 0.078 | H3GA0053804, ALGA0108119, ASGA0044277, ASGA0044267, H3GA0028038, ALGA0054579, ALGA0054588 |
| Block1 | 1212121 | 0.184 | H3GA0053804, ALGA0108119, ASGA0044277, ASGA0044267, H3GA0028038, ALGA0054579, ALGA0054588 |
| Block1 | 2212121 | 0.022 | H3GA0053804, ALGA0108119, ASGA0044277, ASGA0044267, H3GA0028038, ALGA0054579, ALGA0054588 |
| Block2 | 2121112 | 0.525 | ALGA0054598, ALGA0054606, ALGA0054609, ALGA0054577, H3GA0028042, ASGA0044289, ALGA0054619 |
| Block2 | 2222211 | 0.185 | ALGA0054598, ALGA0054606, ALGA0054609, ALGA0054577, H3GA0028042, ASGA0044289, ALGA0054619 |
| Block2 | 1112111 | 0.079 | ALGA0054598, ALGA0054606, ALGA0054609, ALGA0054577, H3GA0028042, ASGA0044289, ALGA0054619 |
| Block2 | 2121222 | 0.204 | ALGA0054598, ALGA0054606, ALGA0054609, ALGA0054577, H3GA0028042, ASGA0044289, ALGA0054619 |
| Block3 | 12 | 0.073 | H3GA0028049, H3GA0041349 |
| Block3 | 22 | 0.079 | H3GA0028049, H3GA0041349 |
| Block3 | 11 | 0.400 | H3GA0028049, H3GA0041349 |
| Block3 | 21 | 0.447 | H3GA0028049, H3GA0041349 |

Additional file 4. Detected happlotypes on the region from 120.5-121.5 Mb on pig chromosome 9

^1^ : 1 is minor allele and 2 is major allele
